# Efficacy of Cannabidiol in Reducing Virulence of *Listeria monocytogenes*

**DOI:** 10.3390/ijms27062682

**Published:** 2026-03-15

**Authors:** Divya Joseph, Leya Susan Viju, Poonam Gopika Vinayamohan, Abraham Joseph Pellissery, Kumar Venkitanarayanan

**Affiliations:** 1 Department of Animal Science, University of Connecticut, Storrs, CT 06269, USA; 2 United States Department of Agriculture, Wyndmoor, PA 19038, USA; 3 Department of Comparative, Diagnostic & Population Medicine, University of Florida, Gainesville, FL 32608, USA

**Keywords:** *Listeria monocytogenes*, cannabidiol, virulence, sub-inhibitory concentration, *Galleria mellonella*

## Abstract

*Listeria monocytogenes* (LM) is a major foodborne pathogen causing illnesses ranging from gastroenteritis to severe systemic infections. The key virulence factors include bacterial motility, hemolysin and lecithinase production, and invasion of host tissues. This study investigated the anti-virulence effects of cannabidiol (CBD), the main non-psychoactive compound in *Cannabis sativa*, against LM. The minimum inhibitory concentration (MIC, 2289 μM; 719.8 µg/mL) and sub-inhibitory concentration (SIC, 11.92 μM; 3.75 µg/mL) of CBD were determined for LM strains Scott A and ATCC 19115. Cultures were treated with SIC, 6× SIC, 1/4× MIC, and MIC to assess effects on motility, hemolysin and lecithinase production, and adhesion and invasion of human intestinal (Caco-2) and brain endothelial (HBMEC) cells, alongside virulence gene expression by RT-qPCR. Cannabidiol’s efficacy was also determined using a *Galleria mellonella* larval infection model at SIC and 6× SIC. Cannabidiol at 6× SIC significantly reduced motility, toxin production, and host cell adhesion and invasion (*p* < 0.05). RT-qPCR revealed downregulation of key virulence genes, including *prfA*, *hly*, *plcA*, *plcB*, *iap*, *motA*, *motB*, *actA*, *inlA*, and *inlB*. In vivo, CBD enhanced larval survival in a dose-dependent manner and cytotoxicity was observed at concentrations above 33.75 µg/mL. These results indicate that CBD, at non-bactericidal levels, effectively suppresses multiple virulence mechanisms in LM, highlighting its potential as a novel anti-virulence agent for food safety and therapeutic applications.

## 1. Introduction

*Listeria monocytogenes* is a Gram-positive, facultative intracellular rod bacteria that causes listeriosis in humans and animals [1]. It is a significant food-borne pathogen that causes the highest number of hospitalizations (>90%) [2] and a mortality rate of around 20% to 30% [3], especially in susceptible individuals, ranking as the third leading cause of food-borne related deaths. Each year, LM causes approximately 1600 illnesses, 1500 hospitalizations, and 260 deaths in the U.S., primarily affecting older adults, pregnant women, newborns, and individuals with weakened immune systems [4,5]. However, listeriosis is a mild and self-resolving disease for those with strong immune systems, typically manifesting as mild gastroenteritis [6]. The psychrotrophic ability of LM coupled with its resistance to both elevated salinity and acidic environments presents a formidable challenge in controlling this pathogen in foods [7,8].

The pathogenesis of LM occurs upon ingesting contaminated food, resulting in gastroenteritis, septicemia, meningitis, and abortion in pregnant women [9,10,11]. Foods such as ready-to-eat meat products [12], soft cheese, unpasteurized milk [13], and fresh produce [14] are high-risk products contributing to listeriosis. Approximately 83% of listeriosis cases are linked to deli meat that is cut and packaged at retail [12,15,16]. The infective dose of listeriosis is estimated to be 10^4^ to 10^7^ cells in immunocompromised people but it is more than 10^7^ in healthy individuals [17,18].

The major virulence factors in LM include its motility, lecithinase activity, hemolysis of RBCs due to the production of listeriolysin O (LLO), and its ability to attach and colonize the intestinal and brain cells. Internalins [19,20], LLO [21,22], phospholipases [23,24], and actin polymerization protein (ActA) [25,26] help in the attachment, vacuolar escape, intracellular proliferation and cell–cell spread of the bacterium. Antibiotics are the drug of choice for treating LM infection, but LM has gained resistance to a wide range of antibiotics commonly used to treat listeriosis [27,28,29,30].

The treatment of listeriosis is primarily guided by antimicrobial susceptibility profiles of LM, with β-lactam antibiotics forming the cornerstone of therapy. Agents such as penicillin, ampicillin, and amoxicillin are widely used, either as monotherapy or in combination with an aminoglycoside, most commonly gentamicin, to enhance bactericidal activity [31,32,33,34,35]. In severe clinical presentations, including listerial meningitis, adults with normal renal function are typically treated with high-dose ampicillin administered at 2 g intravenously every 4–6 h, or penicillin G at 4 million units intravenously every 4 h, in combination with gentamicin at 1.7 mg/kg intravenously every 8 h for a minimum duration of three weeks [35]. When β-lactam antibiotics are contraindicated due to reduced susceptibility or patient intolerance, alternative agents with activity against Gram-positive bacteria may be considered. These include tetracyclines, erythromycin, chloramphenicol, vancomycin, and trimethoprim–sulfamethoxazole (TMP/SMX) [32]. TMP/SMX is frequently used in patients unable to tolerate ampicillin and is typically administered at 3–5 mg/kg (trimethoprim component) intravenously every 6 h for at least three weeks [32,35,36,37]. Vancomycin is often reserved for cases of LM bacteremia, while erythromycin provides an additional option for individuals who cannot receive ampicillin and/or gentamicin [32,38]. More recently, fluoroquinolones such as levofloxacin have demonstrated activity against LM in vitro and in animal infection models, suggesting potential utility under specific clinical circumstances [34,35]. Treatment considerations become particularly complex during pregnancy, where both maternal outcomes and fetal safety must be carefully balanced. In pregnant patients with listeriosis, ampicillin or erythromycin administered intravenously, or oral amoxicillin, is commonly prescribed for a minimum of 14 days and may be continued until delivery if clinically indicated [18,39]. In cases of penicillin intolerance, TMP/SMX is generally recommended; however, because trimethoprim may exert teratogenic effects during early gestation, erythromycin is preferred in pregnant patients due to its established safety profile for the fetus [18,40].

While LM is traditionally regarded as susceptible to several antimicrobials commonly used to treat Gram-positive infections, including β-lactams, gentamicin, erythromycin, tetracycline, rifampicin, and vancomycin, effective clinical management is increasingly challenged by both intrinsic and acquired resistance mechanisms [18,41,42]. The organism is inherently resistant to cephalosporins, nalidixic acid, and polymyxin E, and many isolates exhibit reduced susceptibility to fluoroquinolones, third- and fourth-generation cephalosporins, fosfomycin, oxacillin, and lincosamides, thereby limiting therapeutic options [18,41,43]. In addition, elevated levels of resistance to tetracyclines have been reported in certain strains, further complicating treatment strategies [41,44]. Importantly, antimicrobial susceptibility profiles of LM are highly heterogeneous and influenced by geographic origin, source of isolation, and temporal factors, reflecting the dynamic and evolving nature of resistance patterns [18]. The growing clinical relevance of antimicrobial resistance is further highlighted by the emergence of multidrug-resistant (MDR) LM strains. The first MDR strain of human origin was identified in France in 1988 and demonstrated resistance to multiple antibiotic classes, including chloramphenicol, erythromycin, streptomycin, and tetracycline, with resistance genes located on a plasmid [45]. Since this initial report, additional MDR isolates have been recovered from clinical, food, and environmental sources across diverse geographic regions, underscoring the pathogen’s ability to acquire and disseminate resistance determinants via mobile genetic elements [33,44,46,47,48,49,50,51]. Taken together, the increasing prevalence of antimicrobial resistance, combined with the severe clinical consequences of listeriosis, underscores the urgent need to pursue novel antibiotics as well as complementary therapeutic strategies beyond conventional bactericidal approaches.

The growing challenge of antimicrobial resistance in LM has prompted increased interest in non-conventional strategies to manage infection beyond standard antibiotic therapy [18,52]. Consequently, increasing attention has been directed toward plant-derived antimicrobial compounds as alternative or adjunct agents. These natural products act through multiple mechanisms, such as compromising bacterial membrane structure, altering permeability, or interfering with efflux systems essential for bacterial survival [18,53]. Several plant-derived chemical compounds have been extensively documented for their inhibitory activity against LM, providing strong justification for exploring natural alternatives to conventional antimicrobials [54]. Compounds such as trans-cinnamaldehyde from cinnamon, eugenol from clove, thymol and carvacrol from thyme and oregano, citral from lemongrass, and oleuropein from olive have been shown to effectively suppress LM growth and survival in vitro [55,56,57,58]. Terpenoid compounds, including limonene and carvacrol, have shown notable activity against LM [59,60]. Beyond growth inhibition, sub-inhibitory concentrations of several phytochemicals significantly downregulated virulence-associated genes involved in motility, toxin production, adhesion, and host cell invasion, resulting in reduced hemolytic activity, impaired motility, and diminished epithelial cell adhesion and invasion [22,58,61,62]. Importantly, attenuation of virulence by these compounds has been validated in biologically relevant infection models, including human intestinal and endothelial cell lines, where treatment with trans-cinnamaldehyde, carvacrol, thymol, or eugenol markedly improved host survival outcomes [58,61,62,63]. Moreover, studies using the *Galleria mellonella* infection model have revealed that phytochemicals such as trans-cinnamaldehyde, carvacrol, and thymol can significantly attenuate LM virulence rather than solely inhibiting growth [63]. Although these findings highlight the therapeutic potential of natural antimicrobial compounds, their clinical application for the treatment of listeriosis remains dependent on further validation in mammalian models and human studies [53]. From a translational perspective, numerous studies have demonstrated that plant-derived compounds can be successfully incorporated into food-relevant delivery systems, such as active packaging films, edible coatings, marinades, and encapsulated formulations, leading to substantial reductions of LM in meat, dairy, produce, and ready-to-eat food models during storage [64,65,66,67,68]. The demonstrated feasibility of plant-derived antimicrobials and antivirulence compounds provides a strong conceptual basis for evaluating CBD as a natural approach to reduce LM pathogenicity. While plant-based compounds have been explored for food-related applications, the present study does not merely address the incorporation of CBD into food delivery systems; rather, it also explores on elucidating its potential role in controlling LM infection in the context of clinical regimen.

Historically, plants have contributed to the development of novel drugs and served as active components in a number of herbal and traditional medicines [69]. Many plant-derived chemicals have previously been shown to possess significant antibacterial properties against Gram-positive and Gram-negative bacteria [61,70,71,72,73,74,75,76]. Cannabidiol (C_21_H_30_O_2_) (CBD) is a non-psychoactive plant-derived compound obtained from the plant *Cannabis sativa*. It is reported to possess antimicrobial properties against a wide range of microorganisms, especially Gram-positive bacteria [77,78,79,80,81,82]. Wassmann et al. (2020) reported that CBD is an effective adjuvant in combination with bacitracin for killing Gram-positive bacteria [78]. Cannabidiol has neuroprotective properties [83], including the blood–brain barrier (BBB) [84,85]. It is also reported to exert protective effects on the intestinal barrier [86,87]. The FDA in 2018 and the European Medicines Agency (EMA) in 2019 approved a pure oil-based liquid formulation of CBD, known as Epidiolex^®^ and Epidyolex, respectively for the oral management of two epilepsy conditions, namely Dravet syndrome and Lennox–Gastaut syndrome [80]. The application of CBD as an ingredient in food and health supplements has been increasing in recent years, especially driven by increasing consumer interest in natural wellness products [88].

Cannabidiol has been reported to exert antibacterial activity through multiple, primarily membrane-associated mechanisms. Evidence from radiolabeled macromolecular synthesis assays demonstrates that CBD rapidly inhibits protein, DNA, RNA, and peptidoglycan synthesis at concentrations near the minimum inhibitory concentration, consistent with a membrane-targeted bactericidal effect rather than inhibition of a single biosynthetic pathway [80]. This membrane-disruptive activity is further supported by observations of membrane depolarization, bacterial cytological profiling characteristic of membrane-permeabilizing agents, and rapid uptake of the normally membrane-impermeable SYTOX™ Green dye, collectively indicating loss of cytoplasmic membrane integrity and function [80]. In addition to direct membrane disruption, CBD has been shown to modulate bacterial pathogenicity by inhibiting membrane vesicle release and altering vesicle protein composition in Gram-negative bacteria, thereby enhancing the bactericidal activity of selected antibiotics and functioning as a potential antibiotic adjuvant [89]. Moreover, studies in Gram-positive bacteria have demonstrated that CBD damages bacterial cell walls and membranes, leading to widespread disruption of metabolic and biosynthetic pathways and global alterations in proteomic and metabolomic profiles, further contributing to its antibacterial activity [90].

The objective of this study was to investigate the efficacy of CBD in attenuating the major virulence factors in LM in vitro and controlling listeriosis in the invertebrate model, *G. mellonella*.

## 2. Results

### 2.1. Effect of CBD on the Growth of LM

To evaluate the effect of CBD on the growth of LM, bacterial cultures were exposed to increasing concentrations of CBD in tryptic soy broth and incubated at 37 °C for 24 h, after which bacterial growth was quantified by CFU enumeration to determine the sub-inhibitory concentration (SIC) and minimum inhibitory concentration (MIC). In this study, the SIC of CBD was defined as the highest concentration that did not result in a statistically significant reduction in LM growth relative to the control but had an effect on the transcription of genes while MIC was defined as the lowest concentration of CBD that completely inhibited bacterial growth, yielding CFU counts comparable to the initial inoculum at 24 h.

**Figure 1:**
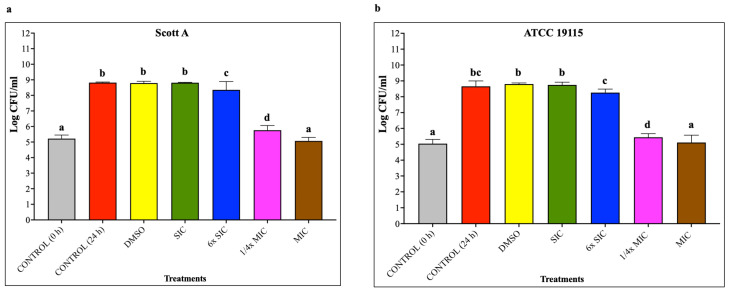
(**a**) Effect of cannabidiol on growth of *L. monocytogenes* Scott A after 24 h incubation at 37 °C. (**b**) Effect of cannabidiol on growth of *L. monocytogenes* ATCC 19115 after 24 h incubation at 37 °C. *L. monocytogenes* (~5 log CFU) was grown in tryptic soy broth with or without different concentrations of CBD for 24 h at 37 °C, and viable counts were determined by serial dilution and plating on TSA and Oxford agar plates. Bars show mean log CFU/mL ± SD (n = 9). Different superscript letters indicate significant differences observed with treatments compared to the control (*p* < 0.05). Cannabidiol concentrations used were: SIC = 11.92 μM (3.75 µg/mL), 6× SIC = 71.52 μM (22.5 µg/mL), 1/4× MIC = 572.25 μM (179.95 µg/mL), and MIC = 2289 μM (719.8 µg/mL). The final concentration of DMSO (100% stock) was 1.2% (*v*/*v*). Abbreviations: DMSO, dimethyl sulfoxide (solvent control); SIC, sub-inhibitory concentration; MIC, minimum inhibitory concentration; CFU, colony-forming units.

Using these criteria, the SIC and MIC of CBD against LM Scott A and ATCC 19115 were found to be the same. The SIC, 6× SIC, 1/4× MIC and MIC against LM were 11.92 μM (0.000375% *w*/*v*; 3.75 µg/mL), 71.52 μM (0.00225%; 22.5 µg/mL), 572.16 μM (0.018%; 180.0 µg/mL) and 2289.56 μM (0.072%; 719.8 µg/mL), respectively. The effect of various concentrations of CBD on LM growth is depicted in Figure 1. Cannabidiol demonstrated a concentration-dependent inhibitory effect LM with no significant reductions in bacterial counts (*p* < 0.05) at SIC and 6× SIC. A minimal reduction of less than 0.5 log CFU/mL in LM population was observed at 6× SIC, whereas a 3 log CFU/mL reduction was noted at 1/4× MIC of CBD (*p* < 0.05). As expected, the MIC of CBD completely inhibited LM growth yielding counts similar to control (no CBD) at 0 h of incubation (inoculation level) (*p* < 0.05), indicating a bacteriostatic effect.

### 2.2. Motility Assay

**Figure 2:**
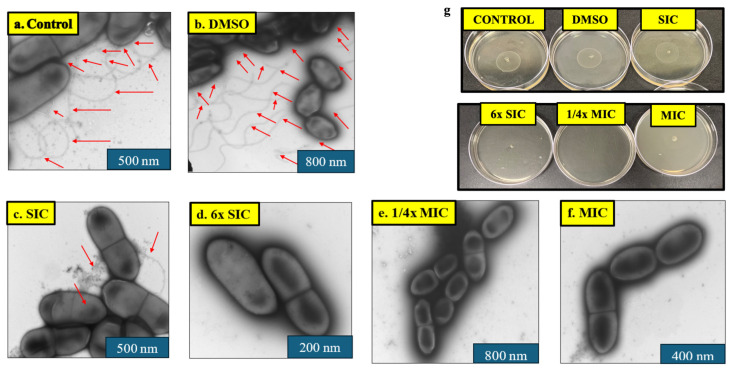
(**a**–**f**) Effect of cannabidiol on the morphology and external structures (flagella) of LM Scott A observed by negative staining transmission electron microscopy (TEM). *L. monocytogenes* cultures grown in the presence or absence of CBD were fixed, negatively stained with uranyl acetate, and examined under the transmission electron microscope to assess flagellar structure. Control and DMSO-treated LM cells exhibited numerous long flagella (indicated by red arrows). In contrast, cells exposed to the subinhibitory concentration (SIC) of cannabidiol showed a marked reduction in flagellar development. Complete loss of flagella was observed at higher concentrations (6× SIC to MIC), indicating a dose-dependent inhibitory effect of cannabidiol on flagellar synthesis. Scales are expressed in nm. (**g**) Effect of cannabidiol on the motility of LM Scott A. A complete inhibition in motility was observed from 6× SIC to MIC. The final concentration of DMSO (100% stock) was 1.2% (*v*/*v*). Abbreviations: DMSO, dimethyl sulfoxide (solvent control); SIC, sub-inhibitory concentration; MIC, minimum inhibitory concentration.

Given the role of motility in LM virulence, the effect of CBD on bacterial motility was assessed by stab inoculation of LM onto semi-solid LB agar plates containing CBD, followed by incubation at 37 °C for 12 h. The effect of CBD on LM motility is given in Table 1. A dose-dependent reduction in the zone of LM motility was observed both isolates. Plates containing no CBD (control) and DMSO (solvent control) showed an average motility of zone of 2.85 cm. The SIC of CBD did not decrease LM motility (*p* > 0.05) whereas further increasing levels of CBD starting from 6× SIC completely inhibited the motility (*p* < 0.05) compared to the control (Figure 2g).

### 2.3. Negative Staining TEM

To determine whether CBD alters LM morphology and flagellar structures, LM cultures grown overnight at 37 °C in the presence or absence of CBD were examined using negative staining transmission electron microscopy. Peritrichous flagella were observed on LM in the control and solvent control treatments (Figure 2a–f). However, a marked reduction in the number of flagella was noticed at the SIC of CBD. Interestingly, no flagella were observed on LM from 6× SIC to MIC. Additionally, no morphological changes were detected on the cellular structure from low to high CBD concentrations.

### 2.4. Hemolysis Assay

**Figure 3:**
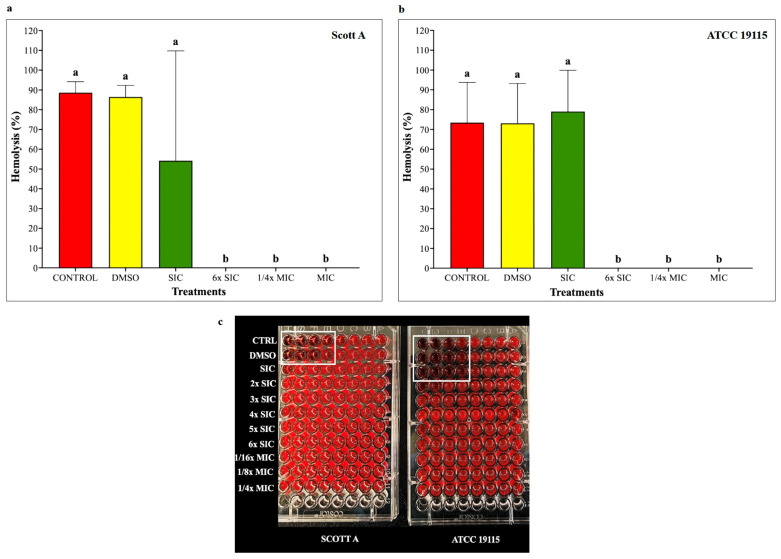
(**a**) Effect of cannabidiol on the hemolysis of *L. monocytogenes* Scott A. (**b**) Effect of cannabidiol on the hemolysis of *L. monocytogenes* ATCC 19115. (**c**) Representative microplate images demonstrating red blood cell lysis at different cannabidiol treatments (from SIC to 1/4× MIC), showing reduced hemolysis at higher CBD concentrations (white boxes indicate hemolyzed wells). Hemolytic activity was determined by measuring listeriolysin O activity in culture supernatants of LM grown for 12 h at 37 °C in the presence or absence of cannabidiol (SIC, 6× SIC, 1/4× MIC, and MIC). The 12 wells containing bacterial supernatants are labeled according to treatment groups. Supernatants were serially diluted in PBS from left to right (H to A). Supernatants were incubated with 3% sheep red blood cells and hemolysis was quantified spectrophotometrically at 600 nm. Hemolysis was visually identified as clear, transparent wells indicating red blood cell lysis, whereas non-hemolyzed wells appeared bright red with intact RBC sedimentation. In Scott A, visible hemolysis in this representative replicate was observed only in the Control and DMSO groups, whereas in ATCC 19115, hemolysis was observed in the Control, DMSO, and SIC groups. No visible hemolysis was detected at higher CBD concentrations. Percent hemolysis was calculated relative to positive (distilled water, 100% lysis) and negative (PBS, 0% lysis) controls. Bars represent the mean hemolysis (%) ± SD (n = 6). Different superscript letters indicate significant differences across treatments compared to the control (*p* < 0.05). Abbreviations: DMSO, dimethyl sulfoxide (solvent control); SIC, sub-inhibitory concentration; MIC, minimum inhibitory concentration.

To assess the impact of CBD on listeriolysin O–mediated hemolytic activity, LM cultures were grown for 12 h at 37 °C with or without CBD, and hemolysis was quantified using sheep red blood cells. Hemolysis of 90% and 70% was produced by LM in controls (Figure 3). The hemolytic activity of LM treated with the SIC of CBD was not different from controls (*p* > 0.05). However, CBD starting from 6× SIC of CBD completely inhibited the hemolytic activity in both isolates.

### 2.5. Lecithinase Plate Test

**Figure 4:**
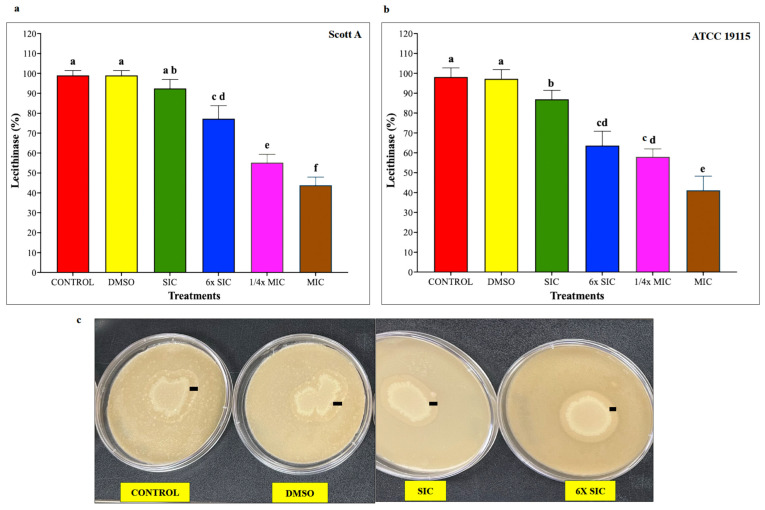
(**a**) Effect of cannabidiol on the lecithinase activity of *L. monocytogenes* Scott A. (**b**) Effect of cannabidiol on the lecithinase activity of *L. monocytogenes* ATCC 19115. (**c**) Representative images of lecithin agar plates demonstrating reduced opalescence in cannabidiol-treated samples compared to control and DMSO groups. *L. monocytogenes* was spot inoculated on to lecithin plates prepared with or without CBD and incubated at 37 °C for 72 h. Lecithinase activity was quantified by measuring the diameter of the opalescent precipitation zone (mm, indicated as small black lines) surrounding bacterial growth. Bars represent the mean lecithinase activity (%) ± SD (n = 6). Different superscript letters indicate significant differences across treatments compared to the control (*p* < 0.05). Abbreviations: DMSO, dimethyl sulfoxide (solvent control); SIC, sub-inhibitory concentration; MIC, minimum inhibitory concentration.

Because lecithinase activity is an important virulence determinant of LM, lecithinase production was evaluated following incubation of LM on lecithin-containing agar plates at 37 °C for 72 h in the presence or absence of CBD. Lecithinase activity of LM was measured as a zone of opalescence (mm). The relative dose-dependent percentage reduction in lecithinase activity by CBD in the two isolates is depicted in Figure 4. A significant reduction in lecithinase activity was observed from 6× SIC of CBD in Scott A and from SIC in LM ATCC 19115 (*p* < 0.05), while a greater than 50% decrease was observed in samples treated with the MIC of CBD.

### 2.6. Adhesion and Invasion Assays on Caco-2 Cells and HBMEC

**Figure 5:**
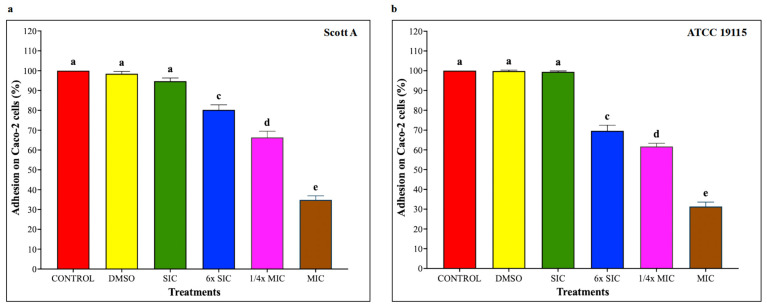
(**a**) Effect of cannabidiol on the adhesion of *L. monocytogenes* Scott A onto Caco-2 cells. (**b**) Effect of cannabidiol on the adhesion of *L. monocytogenes* ATCC 19115 onto Caco-2 cells. *L. monocytogenes* cultures (~7 log CFU/mL) pre-exposed to CBD for 5 h were used to infect Caco-2 monolayers (Multiplicity of Infection (MOI) of 1:100) for 1 h, followed by washing, cell lysis, and enumeration of adherent bacteria by plating. Bars represent the mean adhesion on Caco-2 cells (%) ± SD (n = 6). Different superscript letters indicate significant differences across treatments compared to the control (*p* < 0.05). Abbreviations: DMSO, dimethyl sulfoxide (solvent control); SIC, sub-inhibitory concentration; MIC, minimum inhibitory concentration.

**Figure 6:**
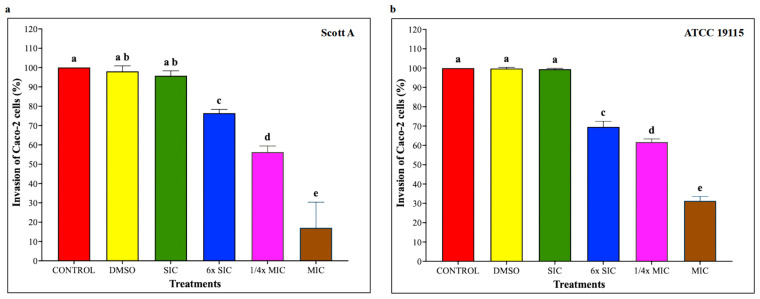
(**a**) Effect of cannabidiol on the invasion of *L. monocytogenes* Scott A into Caco-2 cells. (**b**) Effect of cannabidiol on the invasion of *L. monocytogenes* ATCC 19115 into Caco-2 cells. *L. monocytogenes* cultures grown in the presence or absence of CBD (~7 log CFU/mL) were used to infect confluent Caco-2 monolayers for 1 h. Following washing to remove non-adherent bacteria, monolayers were incubated for 2 h in medium containing gentamicin (100 μg/mL) to eliminate extracellular bacteria. Cells were then lysed, and intracellular bacteria were quantified by serial dilution and plating. Bars represent the mean adhesion on Caco-2 cells (%) ± SD (n = 6). Different superscript letters indicate significant differences across treatments compared to the control (*p* < 0.05). Abbreviations: DMSO, dimethyl sulfoxide (solvent control); SIC, sub-inhibitory concentration; MIC, minimum inhibitory concentration.

**Figure 7:**
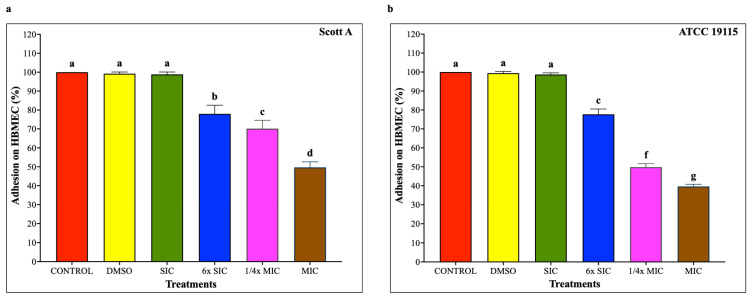
(**a**) Effect of cannabidiol on the adhesion of *L. monocytogenes* Scott A onto HBMEC. (**b**) Effect of cannabidiol on the adhesion of *L. monocytogenes* ATCC 19115 onto HBMEC. *L. monocytogenes* cultures (~7 log CFU/mL) pre-exposed to CBD for 5 h were used to infect HBMEC monolayers (MOI 100) for 1 h, followed by washing, cell lysis, and enumeration of adherent bacteria by plating. Bars represent the mean adhesion on HBMEC (%) ± SD (n = 6). Different superscript letters indicate significant differences across treatments compared to the control (*p* < 0.05). Abbreviations: DMSO, dimethyl sulfoxide (solvent control); SIC, sub-inhibitory concentration; MIC, minimum inhibitory concentration.

**Figure 8:**
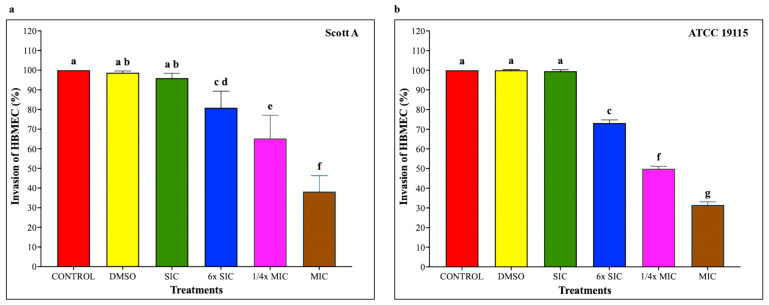
(**a**) Effect of cannabidiol on the invasion of *L. monocytogenes* Scott A into HBMEC. (**b**) Effect of cannabidiol on invasion of *L. monocytogenes* ATCC 19115 into HBMEC. *L. monocytogenes* cultures grown in the presence or absence of CBD (~7 log CFU/mL) were used to infect confluent HBMEC monolayers for 1 h. Following washing to remove non-adherent bacteria, monolayers were incubated for 2 h in medium containing gentamicin (100 μg/mL) to eliminate extracellular bacteria. Cells were then lysed, and intracellular bacteria were quantified by serial dilution and plating. Bars represent the mean adhesion on HBMEC (%) ± SD (n = 6). Different superscript letters indicate significant differences across treatments compared to the control (*p* < 0.05). Abbreviations: DMSO, dimethyl sulfoxide (solvent control); SIC, sub-inhibitory concentration; MIC, minimum inhibitory concentration.

To investigate whether CBD affects LM host–cell interactions, adhesion and invasion assays were performed on Caco-2 cells and human brain microvascular endothelial cells (HBMEC) using LM pre-exposed to CBD for 5 h, followed by 1 h infection for adhesion and additional 2 h for invasion, at a multiplicity of infection of 100 and subsequent incubation under standard assay conditions. As shown in Figure 5, CBD starting from 6× SIC resulted in a dose-dependent reduction in LM attachment to Caco-2 cells, where bacterial adhesion was decreased by approximately 20%, 30% and 65% in both isolates compared to controls by 6× SIC, 1/4× MIC and MIC, respectively (*p* < 0.05). Similarly, LM invasion of Caco-2 cells (Figure 6) was also reduced by 6× SIC, 1/4× MIC and MIC CBD compared to controls (*p* < 0.05). The effect of CBD on LM adhesion and invasion of HBMEC is depicted on Figure 7, and Figure 8, respectively. A concentration-dependent reduction in LM adhesion and invasion of HBMEC (*p* < 0.05) was observed, where the MIC of CBD decreased LM populations by more than 50% compared to control.

### 2.7. RT-qPCR

**Figure 9:**
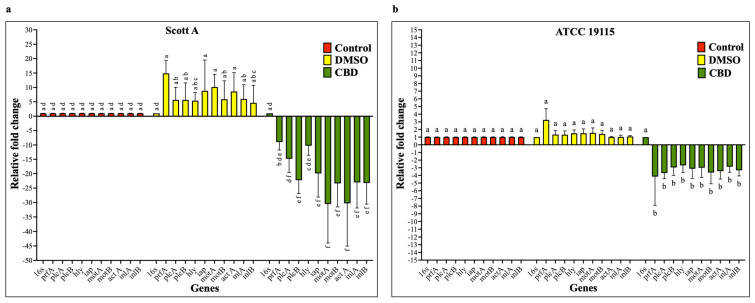
(**a**) Effect of cannabidiol on the transcription of major virulence genes of *L. monocytogenes* Scott A. (**b**) Effect of cannabidiol on the transcription of major virulence genes of *L. monocytogenes* ATCC 19115. Gene expression analysis was performed by RT-qPCR on bacterial cultures grown for 24 h in the presence or absence of CBD at SIC, with relative expression levels calculated using the comparative Ct (ΔΔCt) method and normalized to 16S rRNA as the endogenous control. Bars represent the mean relative fold change ± SD (n = 4). Different superscript letters indicate significant differences across treatments compared to the control (*p* < 0.05). Abbreviations: CBD, cannabidiol; DMSO, dimethyl sulfoxide; SIC, sub-inhibitory concentration.

To determine whether CBD modulates the transcription of key LM virulence genes, bacterial cultures were grown for 24 h at 37 °C with the SIC of CBD, after which total RNA was isolated, and gene expression was analyzed by RT-qPCR. The effect of CBD on the transcription of virulence genes is given in Figure 9. The RT-qPCR analysis revealed that the SIC of CBD significantly down-regulated all tested virulence genes in both LM isolates compared to controls (*p* < 0.05). However, the magnitude of reduction in the transcription of various genes was greater in Scott A compared to ATCC 19115.

### 2.8. Galleria mellonella Survival Assay

**Figure 10:**
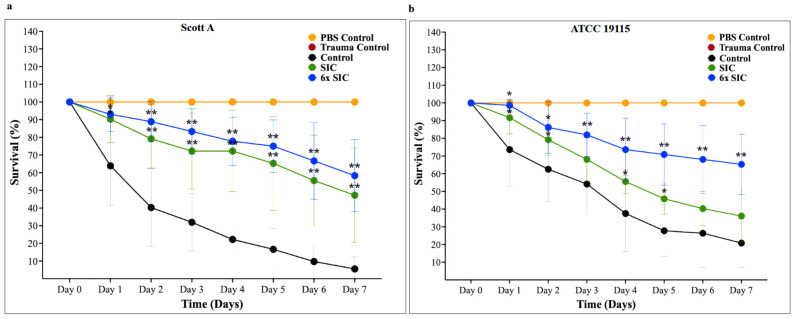
(**a**) Effect of cannabidiol on the survival of *Galleria mellonella* larvae infected with *Listeria monocytogenes* Scott A. Larvae were infected with *L. monocytogenes* and treated with cannabidiol at the sub-inhibitory concentration (SIC) or six-fold SIC (6× SIC). Survival was monitored daily for seven days. Data points represent mean survival percentage ± SD (n = 6). A significant increase in larval survival was observed at both SIC and 6× SIC from Day 1 through Day 7 compared with the untreated infected control (*p* < 0.05). (**b**) Effect of cannabidiol on the survival of *G. mellonella* larvae infected with *L. monocytogenes* ATCC 19115. A significant increase in larval survival was observed at SIC on Days 1, 2, 4, and 5, and at 6× SIC from Day 1 through Day 7 compared with the untreated infected control (*p* < 0.05). Experimental groups included larvae infected with *L. monocytogenes* without treatment (infected control) and larvae infected with *L. monocytogenes* treated with cannabidiol at SIC or 6× SIC. Control groups included larvae maintained under normal experimental conditions without phosphate-buffered saline (PBS), *L. monocytogenes*, cannabidiol, or trauma from needle injury (negative control); larvae administered cannabidiol at 6× SIC in the absence of bacterial infection (CBD control); larvae injected with PBS (PBS control); larvae injected with PBS containing dimethyl sulfoxide (PBS + DMSO control); and larvae subjected to needle puncture only to mimic physical stress (trauma control). These controls were included to distinguish infection-specific mortality from effects related to injection stress, solvent exposure, or cannabidiol toxicity. The trauma control curve overlapped with the PBS control, as both groups exhibited 100% survival throughout the experiment. Other controls, including the CBD control, CBD + DMSO control, and negative control, also showed 100% survival and are not shown in the graph. Statistical analysis was performed using two-way ANOVA followed by Dunnett’s multiple comparisons test, with each treatment group compared to the infected control within the same day (* *p* < 0.05, ** *p* < 0.0001). The SIC and 6× SIC concentrations of CBD were chosen to maintain consistency with the concentrations used across other virulence-related assays in this study, thereby enabling direct cross-comparison of antivirulence effects. Cannabidiol-associated cytotoxicity was observed at concentrations above 9× SIC.

To evaluate the in vivo antivirulence efficacy of CBD, *G. mellonella* larvae were inoculated with LM and treated with CBD at SIC and 6× SIC, larval survival was monitored daily for 7 days post-infection. The effect of CBD on the survival of *G. mellonella* larvae inoculated with LM is shown in Figure 10. The survival rate of LM on day 7 in control samples was ~10% and 25% in Scott A and ATCC 19115, respectively. However, a dose-dependent increase in the survival rate was observed from SIC to 6× SIC in both isolates compared to the control. The survival rate of 6× SIC CBD-treated *G. mellonella* larvae on day 7 was ~65% in Scott A and 70% in ATCC 19115 (*p* < 0.05). The cytotoxic dose of CBD on the larvae was determined to be 9× SIC (33.75 µg/mL).

## 3. Discussion

Given the concern over antibiotic resistance in LM, there is a need for alternative approaches to control this infection. An alternate strategy for managing microbial infections involves the use of anti-virulence agents which target bacterial pathogenicity rather than viability [91]. By attenuating virulence instead of exerting bactericidal pressure, these agents are believed to impose a lower risk of promoting antimicrobial resistance [92,93,94]. In this study, we evaluated the antivirulence efficacy of sub-MICs and MIC of CBD for mitigating listeriosis. Specifically, we investigated CBD’s ability for attenuating the major virulence factors of LM, namely motility, lecithinase and hemolysin production, attachment to invasion of intestinal and brain cells in vitro and control listeriosis in *G. mellonella*. Anti-virulence approaches offer a complementary strategy to conventional antimicrobial therapies by selectively targeting bacterial factors required for host colonization, tissue invasion, and disease progression, rather than directly inhibiting bacterial viability. In the context of LM, anti-virulence agents could be implemented as adjunct interventions aimed at reducing motility, toxin production, and host cell invasion, thereby weakening pathogenic potential and facilitating host immune clearance. Such strategies may be particularly valuable in limiting selective pressure for resistance development, as they do not impose direct bactericidal stress. While further investigation is required to define optimal delivery and therapeutic contexts, the present findings support the feasibility of targeting LM virulence as a practical and resistance-conscious approach to infection control. From a practical perspective, the ability of CBD to suppress LM virulence without complete inhibition of bacterial growth highlights its potential as an anti-virulence agent. Such an approach may complement existing antimicrobial strategies by reducing pathogenicity while minimizing selective pressure for resistance development. Although the present study does not evaluate clinical or food-based delivery systems, these findings provide a conceptual framework for future investigations exploring CBD as an adjunct strategy for mitigating LM infection in human-relevant contexts.

Previously, Marini and coworkers have shown the efficacy of *Cannabis sativa* essential oil against LM, where the major components involved in inhibiting the pathogen were indicated as α-pinene and β-myrcene [95]. However, the US Food and Drug Administration (FDA) removed β-myrcene from the list of approved food additives in 2018 due to the potential to cause liver and kidney tumors [96]. Moreover, cannabis oil has been reported to contain over 400 distinct compounds, including more than 60 cannabinoids, many of which exhibit contrasting physiological effects [97]. Therefore, we selected CBD, a major ingredient in cannabis oil for evaluating its efficacy in controlling listeriosis. Cannabidiol has shown promising potential as an antibacterial agent, particularly against a range of Gram-positive bacteria, including multidrug-resistant bacteria. The efficacy of CBD against Gram-positive bacteria, including drug-resistant genotypes like *Staphylococcus aureus*, *Streptococcus pneumoniae*, and *Clostridioides difficile* was determined by Blaskovich et al. (2021) [80]. At bactericidal concentrations, it inhibited the synthesis of proteins, DNA, RNA, and peptidoglycan in *S. aureus* [80]. In addition, CBD had been shown to selectively reduce specific Gram-negative bacteria, including *Neisseria gonorrhoeae*. However, its effect on Gram-negative bacteria is often limited by the protective outer membrane, which contains lipopolysaccharides [80]. It has shown to reduce *S. mutans* biofilms at 7.5 μg/mL and inhibiting planktonic and biofilm growth with MIC and MBC values [98,99]. Cannabidiol also reduced the metabolic activity of multispecies biofilms and was effective against mature biofilms of *Salmonella typhimurium* and *Enterococcus faecalis* at concentrations as low as 0.125–2 μg/mL, sometimes outperforming standard antibiotics [98,100]. Wassmann et al. (2020) demonstrated that the cyclic peptide antibiotic bacitracin and CBD exhibit potent synergistic antimicrobial activity against Gram-positive bacteria including *Staphylococcus* spp., LM, and *Enterococcus faecalis* [78]. The study reported unusual septa formation and membrane irregularities during cell division, suggesting a potential disruption of the bacterial cell envelope [78]. Furthermore, recent research indicates that topical products that contain active levels of CBG and/or CBD may provide therapeutic benefits for skin conditions such as psoriasis, atopic dermatitis, and acne [101]. Recent studies have demonstrated that CBD can disrupt bacterial membrane integrity, inhibit biofilm formation, and enhance the efficacy of certain antibiotics, suggesting its potential as a novel antimicrobial compound [89,102]. Recent studies have further expanded the understanding of the antibacterial spectrum and mechanism of CBD. Abichabki et al. (2022) demonstrated that although CBD alone shows limited activity against most Gram-negative bacilli, its antibacterial efficacy can be markedly enhanced when combined with polymyxin B, exhibiting additive and synergistic effects even against polymyxin-resistant and extensively drug-resistant *Klebsiella pneumoniae* [103]. Using label-free proteomics and untargeted metabolomics, Zeng et al. reported that CBD induces extensive alterations in protein expression and metabolic pathways in Gram-positive bacteria, affecting hundreds of proteins and metabolites and disrupting cell wall biosynthesis as well as primary and secondary metabolism [90]. Similarly, Sionov et al. (2025) showed that CBD induces membrane hyperpolarization, cytoplasmic ATP leakage, and significant reductions in intracellular metabolic activity in *Gardnerella vaginalis*, along with decreased bacterial survivability and impairment of mature biofilms [104]. These findings provide multi-level mechanistic evidence that CBD exerts antibacterial activity through membrane damage, metabolic dysregulation, and antibiotic potentiation.

In clinical practice, anti-virulence strategies using CBD against LM would be applied by selectively inhibiting key virulence factors or the regulatory pathways either as independent interventions or alongside conventional antibiotic treatments. In the food industry, CBD could potentially be applied as a food additive, surface coatings or on packaging materials for targeting key virulence factors of the pathogen. Additionally, the molecule could also be applied as antibiofilm agents on equipment and food contact surfaces. Hemp-derived CBD is currently incorporated into a range of food products and food supplements, including oils, beverages, confectionery products, bakery items, and nutraceutical formulations, largely owing to its non-psychoactive nature and reported anti-inflammatory, antioxidant, and immunomodulatory properties [88]. These properties position CBD as a compelling candidate for applications in infection control and food safety, where controlling bacterial contamination is critical. Its incorporation into food packaging materials, surface sanitizers, or natural preservatives could offer a plant-derived, non-toxic approach to reducing foodborne pathogens and extending shelf life. However, further research is needed to assess its effectiveness in real-world food systems, regulatory safety, and long-term stability in food matrices.

Motility is a key virulence trait of LM, manifesting through two distinct mechanisms: flagellar motility and actin-based motility. Flagellar motility facilitates bacterial movement within the gastrointestinal tract and is regulated by the *motA* and *motB* genes, which encode essential components of the flagellar motor [105,106]. In contrast, actin-based motility is governed by the *actA* gene, which drives intracellular movement by promoting actin polymerization. *actA* is critical for initiating actin nucleation, filament elongation, and enabling efficient intracellular trafficking, ultimately supporting cell-to-cell spread, bacterial aggregation, and intestinal colonization [107,108]. The loss of motility following CBD treatment appears to arise from direct disruption of flagellar structure and function. Ultrastructural analysis using negative staining transmission electron microscopy revealed a substantial reduction or complete absence of flagellar filaments in CBD-treated cells compared with untreated and vehicle controls, indicating impaired flagellar synthesis or stability. This structural phenotype was further supported by transcriptional analysis, which demonstrated significant downregulation of motility-associated genes, particularly *motA* and *motB*, encoding key components of the flagellar motor stator complex. Notably, *motA* expression was reduced by more than 30-fold in the Scott A strain, suggesting severe impairment of proton motive force–dependent flagellar rotation. Together, these findings indicate that CBD inhibits LM motility through coordinated suppression of flagellar biogenesis and motor function rather than through nonspecific growth inhibition. Given the central role of motility in environmental persistence, surface colonization, and early host interaction, CBD-mediated disruption of motility likely contributes to reduced pathogenic potential and supports its classification as an antivirulence agent.

The adhesion and invasion assays demonstrate that CBD markedly impairs the capacity of LM to adhere to and invade host epithelial and endothelial cells in a dose-dependent manner. These effects are not isolated observations but are supported by complementary structural, functional, and transcriptional data obtained in this study, indicating a coordinated attenuation of multiple virulence determinants. At concentrations ≥ 6× SIC, CBD significantly reduced bacterial adhesion and invasion in both Caco-2 and HBMEC models for Scott A and ATCC 19115 strains. Importantly, these reductions were observed at subinhibitory concentrations that did not substantially affect bacterial viability, suggesting that CBD primarily exerts an anti-virulence effect rather than acting solely through bacteriostatic mechanisms. The impaired host–pathogen interaction is consistent with CBD-induced disruption of bacterial motility. Motility assays showed complete inhibition at 6× SIC and higher concentrations, which was corroborated by negative-stain transmission electron microscopy revealing a pronounced loss of flagellar structures. At the transcriptional level, this phenotype was supported by significant downregulation of motility-associated genes *motA* and *motB*, with motA exhibiting the strongest repression (>30-fold) in the Scott A strain. Given the critical role of flagellar motility in initial surface contact and colonization, its disruption provides a mechanistic explanation for the observed reduction in adhesion. In addition to motility impairment, CBD significantly downregulated genes directly involved in bacterial attachment and invasion, including *inlA*, *inlB*, and *iap*. Internalins A and B are essential for LM entry into non-phagocytic cells, while *iap* contributes to invasion and cell wall remodeling. The coordinated suppression of these genes aligns with the reduced invasion efficiencies observed in both intestinal and blood–brain barrier cell models. Moreover, downregulation of the global virulence regulator *prfA* indicates that CBD acts upstream to broadly suppress virulence gene expression rather than targeting individual effectors.

Upon invading host cells, LM is internalized into phagocytic vacuoles, initiating its intracellular survival and pathogenesis. To facilitate escape and replication within the host, LM employs a suite of virulence factors, notably listeriolysin O (LLO), phospholipase A, and phospholipase B [109]. LLO, a potent pore-forming toxin encoded by the *hly* gene, plays a critical role in lysing red blood cells (RBCs) and disrupting phagocytic vacuoles, thereby enabling LM to escape into the cytosol, replicate, and spread to adjacent cells [110,111]. Beyond its established role in vacuole escape, LLO has been implicated in a range of host–cell interactions, including histone modification and DNA damage response [112,113], immune modulation [114], mitochondrial dynamics [115], and lysosomal disruption [116]. In our hemolysis assay, CBD treatment resulted in a concentration-dependent inhibition of LM hemolytic activity, where a complete inhibition was observed at 6x SIC for both isolates (Figure 2a,b). Given the critical role of hemolysis in LM pathogenesis, these findings suggest that CBD may interfere with LLO production or function, impairing vacuole escape and intracellular LM survival.

*L. monocytogenes* also relies on two phospholipases for vacuolar disruption and cell-to-cell spread. The *plcA* gene encodes phosphatidylinositol-specific phospholipase C (PI-PLC), which collaborates with LLO to degrade the primary vacuole membrane following internalization [117,118,119]. PI-PLC cleaves phosphatidylinositol, generating inositol phosphate and diacylglycerol. The *plcB* gene encodes phosphatidylcholine-specific phospholipase C (PC-PLC), which is essential for dismantling both primary and secondary vacuoles, particularly in the absence of functional LLO [23,119]. PC-PLC also facilitates LM’s cell-to-cell spread. LM-produced phospholipase C can hydrolyze lecithin into phosphorylcholine and insoluble diglycerides [22].

In the lecithinase plate assay, we observed a marked reduction in LM’s lecithinase activity following CBD treatment, evidenced by diminished opaque zones compared to untreated controls (Figure 3a,b). This reduction was apparent at SIC of CBD for ATCC 19115 and starting from 1/32× MIC for both isolates, with a 50–60% decline at MIC. These findings indicate that CBD may disrupt multiple aspects of LM’s intracellular life cycle, highlighting its potential as a therapeutic agent against listerial infections. Cannabidiol also significantly reduced hemolytic and lecithinase activities, reflecting decreased expression or activity of LLO (*hly*) and phospholipases (*plcA* and *plcB*), which are required for vacuolar escape and intracellular survival. Together, the inhibition of motility, adhesion, invasion, and intracellular virulence factors supports a multi-targeted anti-virulence mechanism. Altogether, the adhesion and invasion findings are strongly supported by ultrastructural, functional, and transcriptional evidence demonstrating that CBD disrupts multiple interconnected virulence pathways in LM. Rather than acting solely as a growth inhibitor, CBD compromises bacterial pathogenicity by suppressing flagellar assembly, impairing host cell interaction, and downregulating key virulence regulators and effectors, thereby limiting bacterial colonization and invasion.

The bacterial cell wall plays a critical role in maintaining cell shape, structural integrity, and serving as a barrier against osmotic stress. Therefore, examining structural alterations via electron microscopy can provide valuable insights into the mechanism of action of antimicrobial agents [120]. In this context, microscopic imaging of LM is essential for assessing the impact of CBD on cellular morphology and key virulence structures such as flagella, which facilitate motility. Interestingly, no significant morphological changes were observed in LM cells, even after treatment at elevated concentrations of CBD. However, TEM revealed that CBD treatment inhibited flagellar formation, with noticeable suppression occurring from the SIC up to 1/4× MIC (Figure 8).

Sub-MICs of antimicrobials have been shown to modulate bacterial gene transcription without exerting bactericidal effects [121,122]. In this study, sub-MICs of CBD were employed to investigate their impact on the major virulence determinants of LM. Since these concentrations of CBD do not kill LM, the observed attenuation of virulence in treated samples was not due to bacterial killing but rather suggests a regulatory effect on the transcription of genes associated with LM pathogenesis.

To explore this hypothesis, we conducted RT-qPCR analysis to assess the transcription levels of key virulence genes previously implicated in LM infection in humans. Among these, *prfA* (positive regulatory factor A) encodes a transcriptional activator that positively regulates multiple virulence genes, contributing to motility, adhesion, invasion, and the production of hemolysin and lecithinase [109,123]. The genes *motA* and *motB* are essential for flagellar motor function and bacterial motility, with *motAB* mutants exhibiting impaired movement [124]. Additional invasion-related genes include *inlA*, *intB*, and *iap*, which encode proteins facilitating LM entry into host tissues [125,126,127]. The *hly* gene encodes listeriolysin O, while *plcA* and *plcB* produce phospholipase C enzymes critical for vacuolar escape [128,129]. Following phagosomal escape, LM spreads from cell to cell via the ActA protein, encoded by *actA* [130]. RT-qPCR results demonstrated that CBD significantly downregulated the expression of these virulence genes in both isolates compared to untreated controls (Figure 9a,b).

*G. mellonella*, the larval stage of the greater wax moth, has emerged as a versatile in vivo model for studying microbial pathogenesis across a broad spectrum of organisms, including LM [63,131], *Pseudomonas aeruginosa* [132,133], *Burkholderia cepacia* [134], *Bacillus cereus* [135], *Acinetobacter baumannii* [136], and *Klebsiella pneumoniae* [137]. The innate immune system of *G. mellonella* comprises multiple defense mechanisms, including hemolymph coagulation, cellular phagocytosis, and phenol oxidase-mediated melanization. Pathogen clearance is achieved through mechanisms analogous to those in mammals, such as the action of lysozymes, reactive oxygen species, and antimicrobial peptides like gallerimycin [138].

Given its immunological relevance and experimental tractability, we employed *G. mellonella* as an in vivo model to study the efficacy of CBD in attenuating LM infection. Survival curve analysis revealed that CBD significantly enhanced larval survival compared to untreated controls (*p* < 0.05). Notably, the MIC of CBD increased the survival rate of larvae by more than 60% on day 7 compared to 10–20% survival in controls (Figure 10a,b). These findings suggest that the observed attenuating effect of CBD on LM virulence factors may have played a critical role in protecting the moth from listeriosis.

Some recent studies have demonstrated that CBD exerts antibacterial activity through mechanisms that include disruption of bacterial membrane potential, ATP leakage, and reduction in intracellular metabolic activity. For example, Sionov et al. [104] reported that CBD induces membrane hyperpolarization in *Gardnerella vaginalis*, causes cytoplasmic ATP leakage, reduces intracellular ATP levels, and decreases overall metabolic activity prior to complete loss of viability. Similarly, Barak et al. [139] demonstrated that CBD significantly reduces metabolic activity in *Streptococcus mutans* planktonic cells and biofilms in a dose-dependent manner, as measured by 3-(4,5-dimethyl-2-thiazolyl)-2,5-diphenyl-2H-tetrazolium bromide (MTT) metabolic assay, and that higher concentrations lead to irreversible metabolic suppression. These indicate that CBD can exert metabolic stress before full bactericidal effects are observed. Importantly, in our study, the anti-virulence effects (reduction in motility, hemolysis, adhesion/invasion, and gene expression) were observed at sub-inhibitory concentrations (SIC and 6× SIC), where no significant growth inhibition was detected. This suggests that the attenuation of virulence is not a consequence of bactericidal activity but may reflect metabolic perturbation and altered expression of virulence-associated pathways under sublethal stress conditions. Therefore, while we agree that CBD has documented antimetabolic effects, our results demonstrate that virulence suppression occurs at concentrations that do not inhibit bacterial growth, supporting a true anti-virulence phenotype rather than simple growth inhibition.

Although this study identifies CBD as a promising antivirulence agent with potential therapeutic value, either as a standalone intervention or as an adjunct to conventional antibiotics for the management of listeriosis, several limitations should be acknowledged. The work evaluates CBD effects in only two LM isolates (Scott A and ATCC 19115), so generalizability across additional clinical/food isolates and lineages remains to be confirmed. Morphology assessment used negative-staining TEM (not SEM/HR-SEM), and imaging appears to have been performed on Scott A only; thus, higher-resolution surface imaging and broader isolate-level confirmation of morphology changes were not assessed. In the adhesion/invasion assays, epithelial/endothelial monolayers were infected with CBD-pre-exposed bacteria, rather than treating host cells with CBD during infection; therefore, direct CBD cytotoxicity toward Caco-2/HBMEC under these conditions was not evaluated. In vivo validation is limited to an insect model. Efficacy was assessed in *G. mellonella*, which is a useful screening model but does not fully replicate mammalian intestinal, hepatic, CNS, or fetoplacental infection dynamics relevant to listeriosis. Further studies using diverse clinical isolates and mammalian infection models are warranted to validate its translational relevance and therapeutic applicability.

## 4. Materials and Methods

### 4.1. Bacterial Strains and Cultural Conditions

Two LM strains were used for this study: Scott A and ATCC 19115. Each strain was cultivated separately in 15 mL screw-cap tubes containing 10 mL of sterile tryptic soy broth (TSB) (Difco Becton Dickinson, Sparks, MD, USA) and incubated at 37 °C for 18 h. After incubation, the cultures were diluted serially in sterile phosphate-buffered saline (PBS, pH 7.2) in ten-fold dilutions and 0.1 mL aliquots were plated on duplicate tryptic soy agar (TSA) and oxford agar (Thermo Scientific™ Oxoid™ Listeria Selective Oxford Agar Base (Dehydrated) with Modified Listeria Selective Supplement (Oxford) SR0206)) plates with incubation at 37 °C for 24 h. Bacterial cultures were adjusted to a final concentration of approximately 1 × 10^7^ CFU/mL prior to use in motility, hemolysis, lecithinase, and host cell adhesion and invasion assays. For gene expression analysis, cultures were grown for 24 h prior to RNA isolation, resulting in a final bacterial concentration of approximately 1 × 10^9^ CFU/mL. For the *G. mellonella* assay, the final concentration was 1 × 10^5^ CFU/mL.

### 4.2. Determination of SIC and MIC of Cannabidiol

A 6% solution (*w*/*v*) of cannabidiol (CBD, 98%) (Alfa Biotechnology, Chengdu, China) was made by dissolving CBD powder in 100% dimethyl sulfoxide (DMSO). The SIC and MIC of CBD against LM were determined according to a previously reported method [140,141]. Sterile 15 mL screw-cap tubes containing TSB were inoculated separately with ~5.0 log CFU of LM, supplemented with different concentrations of CBD ranging from 0.0001% to 0.5% (*w*/*v*). Working concentrations were prepared by diluting the CBD stock solution directly into broth to achieve the desired final concentrations. The final DMSO concentration in the MIC treatment group was 1.2% (*v*/*v*), corresponding to the volume of CBD stock added. The solvent control contained an equivalent concentration of DMSO (1.2% *v*/*v*). Lower CBD treatment groups contained proportionally lower final DMSO concentrations according to the dilution factor.

Serial dilutions were plated on TSA and Oxford agars, incubated at 37 °C for 24 h, and the bacterial growth was quantified. The highest concentration of CBD, which did not inhibit bacterial growth after 24 h of incubation was chosen as its SIC, whereas the lowest concentration, which inhibited the growth of LM was selected as the MIC. This experiment used different treatments, including a control without CBD, a solvent control of DMSO (1.2% *v*/*v*), and various concentrations of CBD, including SIC, 1/32× MIC (6× SIC), 1/4× MIC (48× SIC) and MIC (192× SIC). The experiment was conducted three times with duplicate samples.

### 4.3. Motility Assay

The effect of CBD on LM motility was assessed using a previously published method [142]. Separate Luria–Bertani (LB) agar (0.3%) plates were made with the SIC, 6× SIC, 1/4× MIC and MIC of CBD. LB plates prepared without adding CBD served as control and LB plates prepared with DMSO served as DMSO control. Seven log CFU/mL of each LM isolate were stab inoculated into the center of agar plates, incubated at 37 °C for 12 h, and the motility zone was measured.

### 4.4. Negative Staining Transmission Electron Microscopy (TEM)

The external morphology and cellular structures including flagella of LM following CBD treatment was assessed using a transmission electron microscope (TEM), based on previously published protocols [143,144]. An overnight grown culture of LM (Scott A) grown without CBD (control), with DMSO (solvent control), and CBD at SIC, 6× SIC, 1/4× MIC and MIC were subjected to centrifugation at 2000× *g* for 5 min, with subsequent removal of the supernatant. The pellet was then treated with a fixative (1.5% glutaraldehyde + 1.5% paraformaldehyde in 0.12 M phosphate buffer + 3 mM MgCl_2_), pH 7.4 for 15 min, followed by further centrifugation and discarding of the fixative. Dilution with distilled water was performed based on the remaining pellet volume. Briefly, 3 μL of the sample was placed onto a copper-coated 400 mesh grid and air-dried for 2 min. Subsequently, the samples were stained with a 0.5% uranyl acetate solution (100 μL) and blotted away with Whatman filter paper. Once completely dried, the grid was loaded into the sample chamber of a Tecnai T12 G2 Sprite Bio Twin TEM, and images were captured using AMT NanoSprint 12 CMOS camera (Milexia, France).

### 4.5. Hemolysis Assay

The activity of listeriolysin O in the supernatant of LM cultures grown without and with CBD at different concentrations was measured using a hemolysis assay [22,145]. Seven log CFU/mL of LM culture grown for 12 h without CBD and with CBD at SIC, 6× SIC, 1/4× MIC and MIC in 15 mL screwcap tubes was subjected to centrifugation at 12,000× *g* for 10 min. For hemolysis assay, 1 mL syringe-filtered supernatant was collected in 1.5 mL Eppendorf tubes. Defibrinated sheep blood (Fisher Scientific, Agawam, MA, USA) was centrifuged at 600× *g* for 10 min. After removing the supernatant, it was resuspended in PBS. This step was repeated three times until a clear supernatant was obtained. Fresh 3% sheep RBC (SRBC) aliquots were made in PBS (pH—7.4). A volume of 100 μL of LM supernatant, including the control, solvent control, and CBD treatments were serially diluted (2-fold) by mixing with 100 μL of PBS. One hundred microliters of 3% SRBC were added to each well and incubated at 37 °C for 30 min. A positive control (100% hemolysis) was obtained by adding 100 μL of 3% SRBC into 100 μL distilled water, whereas a negative control (0% hemolysis) was obtained by adding the same into 100 μL sterile PBS. After mixing the RBC pellets at the bottom with a vortex mixer, the absorbance was measured at 600 nm. Using the formula, % hemolysis = (1 − ODs/ODt) × 100%, hemolysis was measured where ODs corresponds to the differences in optical density (600 nm) between the sample and positive control, and ODt corresponds to the differences in optical density (600 nm) between the saline control and positive control, respectively [146].

### 4.6. Lecithinase Plate Test

A lecithinase plate test was used to assess the effect of CBD on LM lecithinase production [147]. In brief, Brain heart infusion agar was prepared and lecithin (Fisher Scientific) at 4% (*v*/*v*) was added to the medium stabilized at 45 °C. Cannabidiol was added at SIC, 6× SIC, 1/4× MIC and MIC while dissolving the lecithin. The medium that did not contain CBD served as control. Approximately 7 log CFU/mL of the overnight grown LM was spot inoculated in the center of the plates and incubated at 37 °C for 72 h. The zone of opalescence was measured to analyze the anti-lecithinase activity of CBD. This was expressed in percentage relative to the control.

### 4.7. Cell Culture

The effect of CBD on LM adhesion and invasion of host cells was determined using two different cell lines, namely human enterocyte-like Caco-2 cells (ATCC HTB-27) and human brain microvascular endothelial cells (HBMEC). The cell lines were obtained from ATCC. Caco-2 cells were maintained in MEM (Gibco, Invitrogen, Carlsbad, CA, USA) with 20% fetal bovine serum (FBS, Invitrogen) [148]. The HBMEC were grown in RPMI 1640 (Gibco, Invitrogen) containing 10% FBS (Invitrogen), 10% NuSerum (Becton Dickinson, Bedford, Mass.), 1% MEM vitamin (Invitrogen), 20 mM L-glutamine (Invitrogen), and ten mM sodium pyruvate (Invitrogen) [149]. The cells were seeded onto 24 healthy cell culture plates with a density of ~3 × 10^5^ cells per well and cultured for 24 h at 37 °C in 5% CO_2_.

#### Adhesion and Invasion Assays

The adhesion and invasion assays were performed as previously reported [150]. Approximately 10^5^ cells per well were seeded in 24-well tissue culture plates with whole media and incubated for 18–24 h at 37 °C in a humidified, 5% CO_2_ incubator. An overnight culture of each LM isolate was grown in TSB in 15 mL screw-cap tubes and was exposed to CBD at SIC, 6× SIC, 1/4× MIC and MIC for 5 h. Tubes without any CBD (control) and solvent control (DMSO) were also included. After rinsing the eukaryotic cells with minimal media (MEM for Caco-2 assay and RPMI-1640 for HBMEC assay), they were infected with approximately 7.0 log CFU (MOI of 100) of LM that had been pre-exposed to CBD or left untreated.

For the adhesion assay, the infected monolayers were rinsed three times in PBS after 1 h of incubation, and the cells were lysed with 0.1% Triton X-100. The number of viable adherent LM was determined by serial dilution and culturing on TSA and oxford agar plates. For the invasion assay, the monolayers were incubated for 1 h following infection, rinsed three times in minimal media and incubated for another 2 h in whole media with 10% FBS containing gentamicin (100 μg/mL) (Invitrogen) to kill the extracellular bacteria. The wells were then washed with PBS three times, then 1 mL of PBS containing 0.1% Triton X (Invitrogen) was added, followed by incubation at 37 °C with 5% CO_2_ for 15 min to lyse the cells and release the intracellular *Listeria*. The cell lysates were serially diluted, cultured on TSA and Oxford agar plates and incubated at 37 °C for 24 h before counting. The experiments on each cell type were repeated three times in triplicate. The numbers of adherent/invaded LM in control samples were taken as 100% and the numbers of bacteria in the treatments were expressed as a percentage relative to that of the control [140].

### 4.8. RNA Isolation and Real-Time Quantitative PCR (RT-qPCR)

RT-qPCR was used to study the effect of CBD on the expression of LM virulence genes, as previously described [151]. *L. monocytogenes* isolates (Scott A and ATCC 19115) were grown for 24 h with SIC of CBD and without CBD. Total RNA was extracted using the Applied Biosystems™ MagMAX™ Microbiome Ultra Nucleic Acid Isolation Kit with bead tubes (Fisher Scientific). Complementary DNA was synthesized with the iScript cDNA synthesis kit (Bio-Rad) and used as the template for RT-PCR. SYBR Green (Bio-Rad) was used for detecting the PCR amplification products. Table 2 shows the sequence of primers used and the respective genes analyzed. The comparative critical threshold (Ct) real-time PCR system (Applied Biosystems, Carlsbad, CA, USA) was used to determine the relative gene expression. To assess relative gene expression and the effect of CBD on each gene, data were standardized to the endogenous control (16S rRNA), and the amount of candidate gene expression between treated and untreated samples was compared.

### 4.9. In Vivo Galleria mellonella Survival Assay

*G. mellonella* larvae at their final instar stage (weight ~250–300 mg) obtained from a local vendor were kept in a dark environment at room temperature and maintained on wood shavings [152]. These larvae were used within 7 days of shipping. Twelve randomly selected larvae were sacrificed prior to the start of each experiment to ensure that they were free of LM infection. The experiment was conducted according to previously published protocols [58,136,138,153]. Scott A and ATCC 19115 strains of LM were resuspended in sterile PBS at 10^7^ log CFU/mL. The required concentration of CBD was prepared in phosphate-buffered saline (PBS) containing the bacterial suspension. The last-instar larvae were injected with fifty microliters of LM inoculum (10^5^ CFU per larva) along with the controls using a Tuberculin syringe and 25 G (5/8″) needle into the hemocoel through the last left proleg. The negative controls included those groups of larvae which were not inoculated with LM, which consisted of an untreated control, trauma control, PBS control and PBS with DMSO control. The trauma control group consisted of *G. mellonella* larvae subjected to the same handling and injection procedure as experimental groups but injected with needle only, without PBS, bacterial or CBD treatment. This group was included to control for mortality associated with physical injury or injection-related stress rather than infection. The larvae inoculated with LM, but not with CBD was maintained as positive control. The treatments included CBD at SIC and 6× SIC. A CBD control was also included for determining if CBD causes any toxicity to the worms. After injection, the larvae were kept in sterile Petri dishes with multiple vents on sterile bedding material at 37 °C. The live and dead scoring of larvae was done every 24 h for 7 days. When a larva showed no reaction to contact, it was regarded as dead. The experiment was replicated six times independently and the percentage of survival was calculated on each day for 7 days.

### 4.10. Statistical Analysis

Duplicate samples were used in all experiments except with *G. mellonella* and the entire study was repeated three times. In *G. mellonella* trial, each treatment had 12 larvae (N = 96) and the experiment was replicated at least six times. Statistical analyses were performed using GraphPad Prism (version 10.5.0). Data were analyzed using one-way analysis of variance (ANOVA) followed by Tukey’s multiple comparison post hoc test to determine statistically significant differences among treatment groups. For gene expression and *G. mellonella* survival assays, data were analyzed using two-way ANOVA to account for the effects of treatment and experimental condition or time. Dunnett’s multiple comparisons post hoc test was applied to compare each treatment group against the corresponding controls. Results were presented as mean ± standard deviation (SD), and differences were considered statistically significant at *p* < 0.05.

## 5. Conclusions

This study demonstrated that CBD effectively attenuates LM pathogenicity by targeting multiple virulence mechanisms. Furthermore, CBD significantly modulated the expression of *PrfA*, a master regulator of LM virulence and other associated genes, resulting in reduced bacterial invasion, vacuole escape, actin polymerization, and intercellular spread. In vivo assays using *G. mellonella* larvae confirmed CBD’s protective efficacy against LM infection. Collectively, these findings suggest that CBD holds promise as a prophylactic or therapeutic agent, or as an adjunct to conventional antibiotics, in mitigating listeriosis. However, additional follow up studies in an appropriate animal model are warranted to confirm these results and the safety of CBD.

## Figures and Tables

**Table 1:** Effect of CBD on *L. monocytogenes* motility. Different superscript letters within a column indicate significant differences between treatments (*p* < 0.05). The final concentration of DMSO (100% stock) was 1.2% (*v*/*v*).

Treatments	Zone of Motility (cm)	
	SCOTT A	ATCC 19115
CONTROL	2.85 ^a^	2.8 ^a^
DMSO	2.85 ^a^	2.85 ^a^
SIC	2.80 ^a^	2.7 ^ab^
6× SIC	0 ^c^	0 ^c^
1/4× MIC	0 ^c^	0 ^c^
MIC	0 ^c^	0 ^c^

**Table 2:** List of primers and their sequences used for RT-qPCR analysis of *L. monocytogenes* virulence and motility genes.

Genes	Primers	Sequence
rRNA-16S^a^	Forward	5′-TGGCGGACGGGTGAGTA-3′
(NC_012488.1)	Reverse	5′-CCGGAGTTATCCCCAACTTACA-3′
*prfA*	Forward	5′-GCGGTCAACCGTTCCA-3′
(NC_003210.1)	Reverse	5′-TGAGGCTCGTGAGGAATACGA-3′
*plcA*	Forward	5′-TCGGACCATTGTAGTCATCTTGA-3′
(NC_002973.6)	Reverse	5′-CACAAATTCGGCATGCAGTT-3′
*plcB*	Forward	5′-CGCAGCTCCGCATGATATT-3′
(NC_003210.1)	Reverse	5′-GATTATCCGCGGACCAACTAAG-3′
*hly*	Forward	5′-TCTCCGCCTGCAAGTCCTA-3′
(NC_002973.6)	Reverse	5′-TCGATTTCATCCGCGTGTT-3′
*iap*	Forward	5′-CTACAGCTGGGATTGCGGTAA-3′
(NC_003210.1)	Reverse	5′-TGCTTGCGGATGCGATT-3′
*motA*	Forward	5′-CGCTGAAGCTTTAATTGTCATCA-3′
(NC_002973.6)	Reverse	5′-GGGTGCGCCATCATAACAG-3′
*motB*	Forward	5′-TCCATATAGTGATTTGCTGACACTTTT-3′
(NC_003210.1)	Reverse	5′-CGGAACTGGAGGCAAACAGA-3′
*actA*	Forward	5′-CGTCGTCATCCAGGATTGC-3′
(NC_003210.1)	Reverse	5′-TGCTATGGCTTTCCTTCTTTTTTT-3′
*inlA*	Forward	5′-AATGTAACAGACACGGTCTCACAAA-3′
(NC_003210.1)	Reverse	5′-TCCCTAATCTATCCGCCTGAAG-3′
*inlB*	Forward	5′-CGAAAGTACAAGCGGAGACTATCA-3′
(NC_003210.1)	Reverse	5′-GTTTCTGCAAAAGCATCATCTGA-3′

## Data Availability

The data supporting the findings of this study are available from the corresponding author upon reasonable request.

## Abbreviations

CBD	Cannabidiol
LM	*Listeria monocytogenes*
SIC	Sub-Inhibitory Concentration
MIC	Minimum Inhibitory Concentration
DMSO	Dimethyl Sulfoxide
LLO	Listeriolysin O
CFUs	Colony-Forming Units
HBMECs	Human Brain Microvascular Endothelial Cells
TEM	Transmission Electron Microscopy
RT-qPCR	Real-Time Quantitative Polymerase Chain Reaction
PBS	Phosphate-Buffered Saline
